# Subjective health legacy of the Chornobyl accident: a comparative study of 19-year olds in Kyiv

**DOI:** 10.1186/1471-2458-9-417

**Published:** 2009-11-17

**Authors:** Evelyn J Bromet, David P Taormina, Lin T Guey, Joost A Bijlsma, Semyon F Gluzman, Johan M Havenaar, Harold Carlson, Gabrielle A Carlson

**Affiliations:** 1 Department of Psychiatry, Stony Brook University, Stony Brook, NY, USA; 2 New York Medical College, Valhalla, NY, USA; 3 Dianet Dialysis Centra, Academic Medical Centre, Amsterdam, the Netherlands; 4 Ukrainian Psychiatric Association, Kyiv, Ukraine; 5 Altrecht Institute for Mental Health Care, Utrecht, the Netherlands; 6 Department of Medicine, Stony Brook University, Stony Brook, NY, USA

## Abstract

**Background:**

Since the Chornobyl accident in 1986, the physical health of exposed children in Ukraine has been monitored, but their perceived health has not been studied. This study examines health perceptions of Ukrainian adolescents exposed to radioactive fallout *in utero *or as infants, and the epidemiologic and Chornobyl-related influences on self-reported health.

**Method:**

We assessed three groups of 19-year olds in Kyiv: 262 evacuees from contaminated areas near the plant; 261 classmate controls; and 325 population-based controls. The evacuees and classmates were previously assessed at age 11. Structured interviews were conducted with the adolescents and their mothers (N = 766), followed by general physical examinations (N = 722) and blood tests (N = 707). Proportional odds logistic regression and multi-group path analysis were the major statistical tests.

**Results:**

The examination and blood test results were similar across groups except for a significantly elevated rate of thyroid enlargement found by palpation in evacuees (17.8%) compared former classmates (8.7%) and population-based controls (8.0%). In addition, four evacuees and one population control had had a thyroidectomy. Compared to controls, the evacuees rated their health the least positively and reported more medically diagnosed illnesses during the 5 years preceding the interview, particularly thyroid disease, migraine headache, and vascular dystony. The consistent risk factors (p < 0.001) for these subjective health reports were evacuee status, female gender, multiple hospitalizations, and health risk perception regarding Chornobyl. All three groups of mothers rated their children's health more negatively than the adolescents themselves, and maternal ratings were uniquely associated with the adolescents' health reports in the adjusted models. In the longitudinal evacuee and classmate subsamples, path analysis showed that mothers' health ratings when the children were age 11 predicted their later evaluations which in turn were associated with the adolescent self-reports.

**Conclusion:**

The more negative self-evaluations of the evacuees were linked to a number of risk factors, including multiple hospitalizations, health risk perceptions, and epidemiologic risk factors. The increased rate of thyroid cancer and other diagnoses no doubt contributed to the evacuees' less positive subjective health. The strong effect of the mothers' perceptions argues in favor of developing risk communication programs for families rather than for mothers or adolescents as separate target groups.

## Background

The present study examines the health of 19 year old adolescents living in Kyiv, Ukraine, who were exposed to the Chornobyl accident as infants or *in utero*. Since that event, their health has been monitored by local and international agencies. There is incontrovertible evidence of an excess of thyroid cancer in this cohort, and poorly substantiated or inconsistently supported assertions about elevated rates of birth defects, mental retardation, and leukemia [1]. These assertions are frequently and prominently reported in the local media [2]. In light of this attention, it is important to consider how this "Chornobyl generation" came to perceive their health status when they approached young adulthood. Adults sampled from regions contaminated by Chornobyl were significantly more likely to describe their health as unsatisfactory compared to controls [3-5] although no differences were found on physical examination [4]. We hypothesized that a similar discrepancy between objective and subjective health would be found in the cohort raised in the shadow of this event.

In population-based samples of older adolescents, approximately 10% describe their health as unsatisfactory or poor [6-9]. In Ukraine, 13.3% of a national probability sample aged 18-29 rated their health as poor or very poor [10]. Steptoe and Wardle [11] suggested that Eastern European adolescents describe their health less favorably than Western Europeans, but the evidence is contradictory [12,13]. The most consistently found risk factors for less satisfactory health perceptions among adolescents are female gender, poorer relationship with parents, lower educational achievement, lower socio-economic status or parental education, and lower self-esteem [8,9]. As expected, adolescents who have had life threatening medical conditions also report poorer health than controls [14]. A few studies have shown that adolescents rate their health more negatively than their parents [13]. In late adolescence, when parental influence decreases, the two reports are weakly correlated [15]. However, there is insufficient information as to how this relationship varies in different settings and circumstances. In Ukraine, for example, the persistent concerns about the health of the evacuee children could potentially lead to greater parent-child congruence.

In the aftermath of a disaster, especially a toxic event, the risk perceptions that develop become important risk factors for self-rated health in adults [16]. Even though the evacuee teens' experience of the Chornobyl disaster was indirect, the reminders of potential adverse consequences for their health are omnipresent. The exposed children have special health and education benefits, receive periodic check-ups that often take place in the hospital, and are raised in families that are very concerned about their well-being and in particular about the possibility that they could develop thyroid cancer [17]. We thus hypothesized that the adolescents' beliefs about the adverse effects of the accident would be significantly associated with their perceptions of their health status.

Thus, the objectives of this study were to investigate the subjective health of 19 year olds in Kyiv, a group that was raised in the aftermath of the Chornobyl accident, and to examine the epidemiologic and disaster-related risk factors influencing their health reports. The sample also received medical examinations and blood tests to confirm that the study groups were comparable on objective measures of health. We employed the Library of Congress transliteration from Ukrainian for Chornobyl and Kyiv.

## Methods

### Sample

The study was conducted in Kyiv, Ukraine, where thousands of people were relocated after the explosion at the Chornobyl nuclear power station in April 1986. The study took place approximately 19 years after the accident and focused on three groups who were infants or *in utero *when the accident occurred: (1) evacuees from the 30-kilometer zone around the plant, (2) former classmates of these evacuees, and (3) population-based controls.

1. The evacuees were originally selected for study in 1997 from a listing of evacuee families in Kyiv with a child who was *in utero *to age 15 months at the time of the accident [17]. Three hundred evacuees and their mothers participated in the original 1997 survey (response rate = 92%). The children were 11 years old on average. Eighty percent of the evacuee families came from the town of Pripyat, 3 km from the Chornobyl nuclear plant, which was built to house the workers and their families. In 2005-6, when they were 19 years old, 265 evacuees and 243 of their mothers participated again. The only variable that significantly influenced attrition was maternal participation [18].

2. Three hundred Kyiv-born homeroom classmates of the evacuees were selected as a comparison group in 1997 (response rate = 85%). At age 19, 261 of the former classmates participated again, as well as 234 of their mothers. As with the evacuees, the only variable that significantly influenced attrition was mothers' participation [18].

3. A population-based control group was recruited in 2005-6 to provide data on a representative sample of non-evacuee adolescents in Kyiv. They were selected using sampling software at the Kiev International Institute of Sociology that generated a random list of households in Kyiv by first selecting a street, and then an apartment building, and then an apartment at random [18]. Telephone screening (97% of households in Kyiv had telephones) was used to identify eligible non-evacuee households with a child in the same age range as the evacuees who lived in Kyiv in 1997 when the first survey was conducted. The response rates among eligible households were 85.4% for adolescents (327 out of 383) and 79.4% for their mothers (304 out of 383) [18].

### Design and procedure

The study utilized a 2-stage design involving home interviews focused on self-reported health and risk factors followed by medical evaluations at a clinic in Kyiv. Professional interviewers from the Kiev International Institute of Sociology were trained extensively for the study, and 10% of the interviews were monitored directly [18]. The interviews were conducted using a computer-assisted procedure. Stage 2 was conducted by medical staff trained and monitored by Dr. Bijlsma. The adolescents received small cash incentives for their participation and also took part in periodic lotteries [18].

The protocol was approved by the institutional review boards of Stony Brook University, the Kiev International Institute of Sociology (home interviews), and the Ukrainian Psychiatric Association (medical examinations and blood tests). The home interviews when the children were 11 were conducted by interviewers from SOCIS-Gallop of Kyiv, and their IRB approved the protocol at that time. Written informed consent was obtained. All measures were translated from English into Russian and back-translated into English using the World Health Organization guidelines [19]. The Russian version was then translated into Ukrainian.

### Measures

The subjective health measures and risk factors were obtained during the home interview, and the objective medical examination measures were obtained 2-4 weeks later.

#### Subjective health

Participants rated their health on a 5-point scale (excellent, good, satisfactory, poor, very poor). This measure of self-rated health has been widely used in morbidity and mortality research as an overall indicator of health status [20]. Because few participants rated their health as excellent (4.7%) or very poor (0.5%), we combined them for the analysis into a 3 category variable: excellent/good (0), satisfactory (1), and poor/very poor (2).

Self-reports of medically diagnosed and treated illnesses were ascertained from a standard checklist of disorders. The analysis focused on seven conditions often attributed to the Chornobyl accident by the lay public: migraine headaches; anemia; gastrointestinal disease; arthritis; thyroid disease; problems with the immune system; and vascular dystony, a local diagnosis given for non-specific somatic symptoms [21]. A 5-year time frame was used. Scores were trichotomized into 0, 1, and ≥ 2.

#### Risk factors

The epidemiologic risk factors for subjective health were (1) gender, (2) primary role (attending vs not attending university), (3) material circumstances (able to afford some luxuries vs unable), (4) emotional self-esteem (sum of 4 yes/no items selected from Starfield et al. [22]; higher scores indicate lower self-esteem), (5) quality of communication with parents (the higher score of communication with mother [mean of 9 items rated 1 = never to 5 = always; α = 0.84] or father [mean of 4 items also rated 1 = never to 5 = always; α = 0.88] modified from Margolies & Weintraub [23]; higher scores indicate poorer communication), and (6) number of hospitalizations in the past 5 years (coded 0, 1, ≥ 2). We note that the reasons for hospitalization ranged from basic check-ups to minor conditions (sinus problems) to surgical procedures.

Two risk perceptions were also considered, namely, whether participants thought that Chornobyl affected their health and whether they thought that the accident would adversely affect the health of future generations. Possible answers were yes very, yes somewhat, and no. Because approximately two-thirds of the adolescents answered "yes somewhat" and risk perception is meant as a judgment of severity of risk, both variables were recoded as yes very (1) vs somewhat or no (0).

The mothers were asked to assess their children's health on the same two measures described above. The evacuee and classmate mothers also made these ratings when their children were 11 years old. The only difference was that the 1997 medical checklist omitted gastrointestinal disease.

#### Medical examination and blood tests

Basic physical examinations lasting 2-4 hours were conducted at a medical clinic in Kyiv following the home interview. The physicians and nurses were trained in the study protocol by Dr. Bijlsma. They were not told which adolescents were evacuees or controls. The nurses measured body weight and height, temperature, blood pressure, pulse and respiratory rate. Thereafter the individual was examined by an ophthalmologist, cardiologist and internist respectively, in random order. The ophthalmologist examined visual acuity (Sivtsev table and Rott apparatus) and inspected the cornea, pupils, lens, eye chambers and retina (binocular magnifier and electrical ophthalmoscope); the cardiologist examined the heart and blood vessels; the internist completed the physical examination by checking ears, nose, throat, lymph nodes (cervical, axillary, inguinal), thyroid, chest, abdomen, extremities, skin, reflexes and overall nourishment. After the clinic received the results of the blood tests, the physicians also made an overall rating of whether the respondent was healthy, had minor health problems, or had major health problems. This report presents selected results from the medical examination (Table 1).

**Table 1:** Health status of evacuees and controls in Kyiv, Ukraine, at age 19

	Total	Evacuees	Classmates	Population controls
**Medical examination**	**n = 722**	**n = 217**	**n = 229**	**n = 276**
Enlarged lymph nodes	42 (5.8)	14 (6.5)	7 (3.1)	21 (7.6)
Enlarged thyroid^a, b^	80 (8.6)	38 (17.8)	20 (8.7)	22 (8.0)
BMI				
Underweight (<18.5)	87 (12.0)	21 (9.7)	33 (14.4)	33 (12.0)
Normal weight (18.5-24.9)	533 (73.8)	162 (74.7)	167 (72.9)	204 (73.9)
Overweight (25-29.9)	81 (11.2)	27 (12.4)	21 (9.2)	33 (12.0)
Obese (>30)	21 (2.9)	7 (3.2)	8 (3.5)	6 (2.2)
Diastolic blood pressure				
Optimal (<80)	498 (69.0)	143 (65.9)	153 (66.8)	202 (73.2)
Normal (80-84)	175 (24.2)	57 (26.3)	64 (27.9)	54 (19.6)
High (≥ 85)	49 (6.8)	17 (7.8)	12 (5.2)	20 (7.2)
Systolic blood pressure				
Optimal (<120)	471 (65.2)	131 (60.4)	151 (65.9)	189 (68.5)
Normal (120-129)	176 (24.4)	64 (29.5)	57 (24.9)	55 (19.9)
High (≥ 130)	75 (10.4)	22 (10.1)	21 (9.2)	32 (11.6)
Visual acuity, average^c^				
logMAR 0	462 (64.0)	149 (68.7)	142 (62.0)	171 (62.0)
0 < logMAR < 1	208 (28.8)	53 (24.4)	70 (30.6)	85 (30.8)
logMAR ≥ 1	52 (7.2)	15 (6.9)	17 (7.4)	20 (7.2)
Cataracts	4 (0.6)	4 (1.8)	0 (0.0)	0 (0.0)
Physician summary^d^				
Healthy	77 (10.7)	20 (9.2)	35 (15.3)	22 (8.0)
Minor health problem(s)	572 (79.2)	171 (78.8)	177 (77.3)	224 (81.2)
Major health problem(s)	73 (10.1)	26 (12.0)	17 (7.4)	30 (10.9)
				
**Blood tests^e^**	**n = 707**	**n = 213**	**n = 224**	**n = 270**
Hemoglobin (g/dL)				
Median	14.2	14.2	14.0	14.4
Interquartile range	13.2-15.5	13.2-15.3	13.0-15.7	13.3-15.6
Platelets (10^9/L)				
Median	231	231	236	226
Interquartile range	197.0-270.0	195.5-267.0	204.25-272.0	195.75-265.25
Erythrocytes (10^12/L)				
Median	4.8	4.8	4.8	4.8
Interquartile range	4.5-5.2	4.5-5.2	4.4-5.2	4.4-5.2
Leukocytes (10^9/L)				
Median	6.4	6.4	6.5	6.3
Interquartile range	5.4-7.6	5.5-7.6	5.5-7.6	5.3-7.4
TSH^a^				
<0.4	18 (2.6)	4 (1.9)	9 (4.0)	5 (1.9)
0.4-4.0	660 (93.8)	195 (92.9)	207 (92.4)	258 (95.6)
>4.0	26 (3.7)	11 (5.2)	8 (3.6)	7 (2.6)

Number and percentage (in parentheses) of participants having each finding is shown.

^a^Excludes 5 adolescents who had a thyroidectomy (4 evacuees and 1 population control).

^b^Chi squared = 13.7, p < 0.001

^c^LogMAR = logarithm (base 10) of the minimum angle of resolution; average logMAR = average of right and left eye^38^

^d^Chi squared = 9.7, p = 0.046

^e^No differences were found on other blood tests, including differentiation of lymphocytes into B and T cells and NK cells.

Blood samples were drawn from each participant. Erythrocyte sedimentation rate and whole blood smears were prepared immediately on site. Blood samples for hemoglobin, erythrocytes, leukocytes and platelets were examined at the Institute of Experimental Pathology, Oncology and Radiology of the Academy of Sciences of Ukraine using a Sysmex K-1000 Quantitative Automated Hematology Analyzer (TOA Med Electr, Kobe, Japan). Blood samples for TSH (thyroid stimulating hormone) were assayed at the Science and Research Institute of Endocrinology and Metabolism in Kyiv. These samples were stored at 5°C until handling within 24 hours after phlebotomy. Serum was separated by low speed centrifugation (1500 × g) for 15 minutes at room temperature. Plasma was transferred into vials with the help of automatic disposable pipettes (Eppendorf). TSH was estimated by IRMA techniques, using commercial kits from Immunotech Inc. (Beckman, Czech Republic). Measurement of radioactivity, fitting of the standard curve and analysis of samples was carried out using a computerized gamma counter (Beckman 5500B, USA). Assay reliability was determined by the use of commercially derived control sera of low, medium and high concentrations which were included in every run. All assays were carried out in duplicate. The TSH assay had an interassay coefficient of variation of <10%. The normal range for serum TSH, as standardized in the laboratory, was 0.3-4.0 mIU/L.

### Statistical analysis

Three evacuees and two population controls were pregnant at the time of interview and were excluded from the analysis. All statistical analyses were performed in R version 2.7.1 http://www.cran.r-project.org unless otherwise specified. Group differences were examined using chi-squared tests. Proportional odds logistic regression was used to examine the associations of the risk factors with self-rated health coded as excellent/good (0), satisfactory (1), and poor/very poor (2), and number of medical conditions coded as 0, 1, ≥ 2. The proportional odds assumption was checked for all risk factors. Unadjusted and adjusted (for all risk factors) odds ratios and 95% confidence intervals are reported. Interaction terms for the risk factors with group were also tested. Concordance between mothers and children's perceptions was analyzed using a weighted kappa statistic with squared weights [24]. The influence of mothers' perceptions was examined in logistic regression models.

Final path models were conducted with data from the evacuees and classmate controls participating in both assessments points (N = 474). These models integrated the significant risk factors for each subjective health outcome in a multi-group path analysis in Mplus, version 4.1 [25] using the weighted least squares mean and variance (WLSMV) estimator and the theta parameterization. This model was limited to goodness-of-fit indices and included the comparative fit index (CFI) [26] and the root mean square error of approximation (RMSEA) [27]. As a general rule of thumb according to current conventions, adequate fit was defined as a CFI above 0.90 and a RMSEA value less than 0.10 [28,29]. Equality constraints in path coefficients across group were tested with the DIFFTEST procedure, which calculates the change in χ^2 ^of nested models [30].

## Results

### The sample

Eighty-three evacuees (31.7%) were exposed to Chornobyl radiation *in utero*, while 179 (68.3%) were exposed in infancy. The evacuees, classmates, and population controls were similar with regard to gender (51.4% overall was female), primary role (64.6% were attending university), material circumstances (36.5% were unable to afford unnecessary items), level of self-esteem, and quality of family communication. More evacuees (32.8%) than classmates (20.3%) or population controls (18.2%) were hospitalized two or more times in the 5 years preceding the interview (p < 0.001). With regard to risk perceptions, relatively more evacuees believed that their health was very much affected by the accident (19.8% vs 8.8% of classmates and 13.8% of population controls; *p *= 0.005), while similar proportions of the groups thought that the accident would very much affect the health of future generations (15.6% overall).

### Medical examination and blood test results

As shown in Table 1, the overall participation rates in the medical phase of the study were 85.2% for the examination and 83.6% for the blood tests, and the rates did not vary appreciably by group. By and large, the examination and blood test results were similar for the three groups. However, significantly more evacuees were found on palpation to have enlarged thyroids (17.8% vs 8.7% of classmates and 8.0% of population controls; *p *< 0.001). Six participants with thyroid enlargement, two from each group, had nodular thyroids. Serum TSH was similar in individuals with and without thyroid enlargement. We note that four evacuees and none of the controls were found to have cataracts. In addition, based on the physician summary rating, more evacuees (12.0%) and population controls (10.9%) had major health problems compared to the classmate group (7.4%) (*p *= .046).

### Subjective health

The evacuees rated their health less positively than their peers, with 36.3% describing their health as good compared to 52.1% of the classmates and 47.4% of the population controls (Table 2). The evacuees were also more likely to report being diagnosed with 5 of the 7 medical conditions, especially migraine headache, vascular dystony, and thyroid disease (p < 0.001). Overall, 34.0% of evacuees compared to 18.4% of classmates and 21.8% of population controls reported 2 or more illnesses. Self-rated health and number of self-reported illnesses were moderately inter-correlated (phi = 0.33).

**Table 2:** Self-ratings of health and diagnosed illnesses at age 19 in Kyiv, Ukraine

	Total	Evacuees (E)	Classmates (C)	Population Controls (P)		Significant pairwise
	n = 848	n = 262	n = 261	n = 325	χ^2^	comparisons^a^
**Self-rated health**						
Excellent/good	385 (45.4)	95 (36.3)	136 (52.1)	154 (47.4)	16.9 **	E > C*** E> P**
Satisfactory	403 (47.5)	148 (56.5)	112 (42.9)	143 (44.0)		
Poor/very poor	60 (7.1)	19 (7.3)	13 (5.0)	28 (8.6)		
**Illnesses diagnosed in past 5 years**						
Migraine headache	31 (3.7)	19 (7.3)	5 (1.9)	7 (2.2)	13.9 ***	E > C** E> P**
Anemia	27 (3.2)	16 (6.1)	3 (1.1)	8 (2.5)	11.3 **	E > C** E> P*
Vascular dystony^a^	199 (23.5)	90 (34.4)	55 (21.1)	54 (16.6)	26.6 ***	E > C*** E> P***
Gastrointestinal disease	257 (30.3)	97 (37.0)	64 (24.5)	96 (29.5)	9.8 **	E > C**
Arthritis	8 (0.9)	2 (0.8)	3 (1.1)	3 (0.9)	0.2	
Thyroid disease	192 (22.6)	82 (31.3)	42 (16.1)	68 (20.9)	18.1 ***	E > C*** E> P**
Problems with immune system	74 (8.7)	27 (10.3)	18 (6.9)	29 (8.9)	1.9	
**Number of illnesses**						
0	380 (44.8)	88 (33.6)	138 (52.9)	154 (47.4)		E > C*** E> P***
1	260 (30.7)	85 (32.4)	75 (28.7)	100 (30.8)		
2+	208 (24.5)	89 (34.0)	48 (18.4)	71 (21.8)	26.7 ***	

^a^Local diagnosis given for non-specific somatic symptoms such as fatigue, dizziness, and changes in blood pressure.

*p < 0.05 **p < 0.01 ***p < 0.001

Among the evacuees, there were no significant differences in subjective health by time of exposure (*in utero vs*. infancy). The association between self-reported thyroid diagnosis and enlarged thyroid on examination was highly significant (OR = 5.9, 95% CI = 2.8-12.5, p < 0.001). Thus 25 of the 38 evacuees with enlarged thyroid (65.8%) compared to 44 of the 179 evacuees with normal thyroid size (24.6%) reported being diagnosed with thyroid disease in the past. This was expected since the examination included medical history taking.

### Risk factor analysis

Table 3 shows that in the unadjusted analyses, most of the risk factors were significantly associated with self-rated health, apart from university attendance and perceived health risk to future generations due to Chornobyl. None of the interaction terms of group with the risk factors was significant. In the fully adjusted model, female gender, worse material circumstances, poorer self-esteem, hospitalizations, and belief that Chornobyl very adversely affected one's health remained significant. Indeed, those believing that Chornobyl had a very adverse effect on their health (vs somewhat or not) were 3.6 times as likely to rate their health as less satisfactory, and those having ≥ 2 hospitalizations were twice as likely to rate their health as less satisfactory.

**Table 3:** Associations of risk factors with health reports

	Self-rated health (good, moderate, poor)		Self-reported illnesses (0, 1, 2+)	
	OR (95% CI)	aOR (95% CI)	OR (95% CI)	aOR (95% CI)
**Epidemiologic risk factors**				
Group	LRT = 12.6**	LRT = 5.4	LRT = 26.5***	LRT = 15.1***
Evacuee	1.4 (1.01-2.0)*	1.1 (0.8-1.5)	1.8 (1.3-2.4)***	1.6 (1.2-2.2)**
Classmate	0.8 (0.6-1.1)	0.7 (0.5-1)	0.8 (0.6-1.1)	0.8 (0.6-1.1)
Population control	1.0	1.0	1.0	1.0
Female	1.9 (1.4-2.4)***	1.8 (1.3-2.4)***	2.9 (2.2-3.7)***	2.9 (2.2-3.8)***
Not attending university	1.2 (0.9-1.6)	1.1 (0.8-1.5)	0.6 (0.5-0.8)***	0.5 (0.4-0.7)***
Material circumstances	2.1 (1.6-2.8)***	1.7 (1.3-2.3)***	1.5 (1.1-1.9)**	1.2 (0.9-1.5)
Emotional self esteem	1.2 (1.1-1.4)**	1.2 (1.01-1.4)*	1.1 (1.0-1.2)	1.1 (0.9-1.3)
Family communication	1.3 (1.1-1.7)**	1.2 (1.0-1.5)	1.3 (1.1-1.6)**	1.2 (1-1.5)
Number of hospitalizations past 5 years	LRT = 32.4***	LRT = 15.0***	LRT = 72.0***	LRT = 63.0***
None	1.0	1.0	1.0	1.0
One	1.6 (1.2-2.2)**	1.5 (1.1-2.1)*	2.1 (1.5-2.8)***	2.1 (1.5-2.9)***
Two+	2.6 (1.8-3.6)***	2.0 (1.4-2.8)***	3.8 (2.8-5.2)***	3.9 (2.8-5.6)***
**Chornobyl beliefs**				
Chornobyl adversely affected health	4.0 (2.7-6.0)***	3.6 (2.4-5.5)***	4.1 (2.8-5.9)***	2.8 (1.8-4.1)***
Health of future generations affected	0.9 (0.6-1.3)	0.7 (0.5-1)	2.0 (1.4-2.9)***	1.8 (1.2-2.6)**
				
**Model Fit**				
Area under curve (AUC)		0.705		0.732
R^2^		0.170		0.265

*p < 0.05 **p < 0.01 ***p < 0.001

OR = odds ratio aOR = adjusted odds ratio - includes all epidemiologic and Chornobyl variables reported in this table

CI = confidence interval LRT = likelihood ratio test

With regard to number of illnesses, all of the risk factors except family communication were significant in the unadjusted models (Table 3). There were no significant interaction effects of group with the other risk factors. In the fully adjusted model, evacuee status, female gender, attending (vs. not attending) university, hospitalizations, and both of the risk perceptions were significantly associated with reporting more illnesses. Being hospitalized more than once was associated with a 4-fold increase in risk of reporting more medical conditions. Being female and believing that Chornobyl seriously affected one's health elevated the risk ~3-fold.

### Mothers' perceptions of their children's health

We found moderate concordance between the adolescents and their mothers on overall health rating (weighted kappa = 0.22) and number of illnesses (weighted kappa = 0.31). The kappa for self-rated health was somewhat smaller for evacuees (0.15) than classmates (0.22) and population controls (0.26). This pattern was also found for medical conditions (0.18 for evacuees; 0.37 for classmates; 0.29 for population controls). Unlike previous reports indicating that adolescents view their health more negatively than their parents, the reverse occurred in this sample. Specifically, in all three groups, the mothers rated their children's health more negatively and reported more illnesses (p < 0.001).

For the subsample of adolescents whose mothers participated in the study (N = 766), we examined the effect of adding the mothers' rating for the same health variable as a second step in the adjusted models shown in Table 3. We first note that the pattern of risk factor findings shown in Table 3 was replicated before adding the maternal ratings. In the adjusted model for self-rated health, mother's rating of her child's health was highly significant (LRT = 74.3, p < 0.001; aOR for moderate vs good/excellent = 4.3, 95% CI = 2.8-6.7; aOR for poor vs good = 11.2, 95% CI = 6.1-20.5), and the area under the curve increased from 0.71 to 0.75. Similarly, in the analysis of number of illnesses, maternal report was highly significant (LRT = 126.4, p < 0.001; aOR for 1 vs 0 diagnosis = 4.5, 95% CI = 2.9-6.9; aOR for 2+ vs 0 diagnoses = 10.0, 95% CI = 6.5-15.3), and the area under the curve increased from 0.72 to 0.79.

### Longitudinal analysis of evacuees and classmates

**Figure 1:**
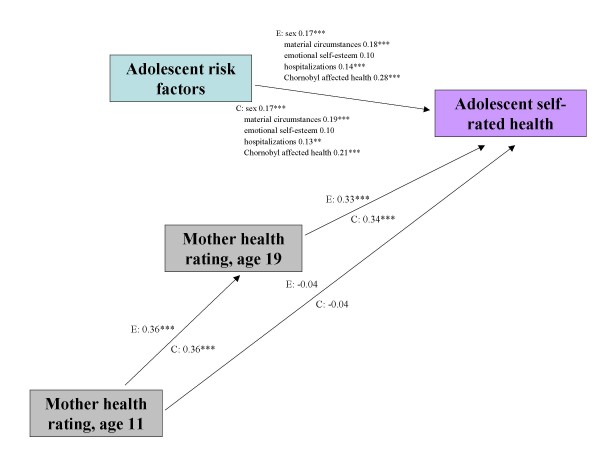
**Path analytic model for adolescent self-rated health in 240 evacuees and 234 classmates in Kyiv**. The estimates shown are standardized estimates and all paths between evacuee and classmate groups have been constrained to be equal. E: Evacuees, C: Classmates. MODEL FIT: Chi-square 25.6, df = 16, p = 0.06; CFI 0.927; RMSEA 0.05. Indirect effects - E: 0.12, p < 0.001 C: 0.12, p < 0.001.

**Figure 2:**
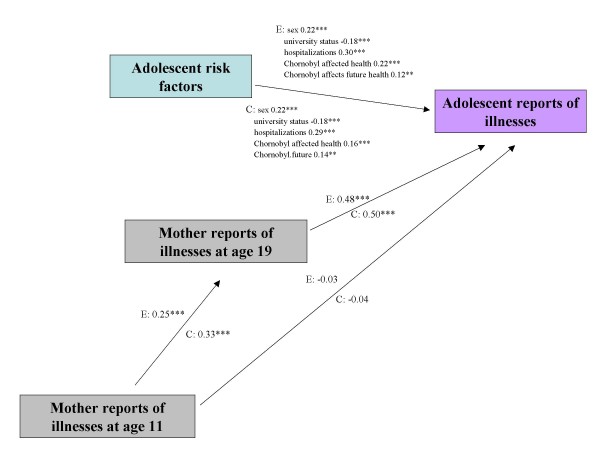
**Path analytic model for adolescent reports of medical illnesses at age 19 in 226 evacuees and 220 classmates in Kyiv**. The estimates shown are standardized estimates and all paths between evacuee and classmate groups have been constrained to be equal. E: Evacuees, C: Classmates. MODEL FIT: Chi-square 39.1, df = 15, p < 0.0001; CFI 0.89; RMSEA 0.08. Indirect effects - E: 0.12, p < 0.001 C: 0.16, p < 0.001.

To examine the role of mothers' ratings of their children's health at age 11, we applied multi-group path analysis (Figures 1 and 2). For each model, we selected the risk factors that were significant in the adjusted analysis for the entire sample (Table 3). The fit of both path models was adequate. For self-rated health (Figure 1), the CFI was 0.93 and the RMSEA was 0.05. For number of medical conditions (Figure 2), the CFI was 0.89 and the RMSEA was 0.08. All paths were constrained to be equal between evacuees and classmates.

Regarding self-rated health, the direct relationships observed in the path model were consistent with the findings from the risk factor analysis (Table 3) and moreover showed that the associations held for both evacuees and former classmates. As expected, there was a moderate correlation between maternal health ratings at age 11 and age 19. Most importantly, there was no direct relationship between mothers' ratings of health at age 11 and adolescents' self-rated health at age 19, but there was an indirect association through maternal health ratings at age 19 (indirect *β *= 0.12, *p *< 0.001). Similarly, for number of illnesses, mothers' report at age 11 was associated with her report of medical diagnoses when the children were 19 but was not directly associated with the adolescents' self-reports. Rather there was a significant indirect effect through mothers' later report (indirect *β *= 0.12 and 0.17 for evacuees and classmates respectively, *p *< 0.001).

## Discussion

To our knowledge, this paper represents the first long-term assessment of the subjective health of youth raised in the shadow of a toxic disaster. Our analysis confirmed the heightened sense of vulnerability about health previously reported in adults following the Chornobyl accident [2-4]. The finding is particularly striking because the sample was by and large healthy, as confirmed by medical examination, and active (two-thirds were attending university). The only physical examination finding that significantly distinguished the evacuee adolescents from controls was an increased prevalence of enlarged thyroid (N = 38) and prior thyroidectomy (N = 4). We also found that four evacuees had cataracts against none of the controls. The most striking risk factors for poorer self-rated health and increased illnesses were female gender, prior hospitalizations, and health risk perceptions. Evacuee status was also associated with illness reports in the adjusted model. None of the risk factors had a synergistic effect with group. Another notable set of findings came from the mothers' health ratings, which were more negative than the adolescent's self ratings and significantly associated with the adolescent reports in the models that adjusted for all of the risk factors. In the panel sample of evacuees and classmates, mothers' health perceptions when the children were 11 predicted their health ratings for age 19 but did not directly influence the self reports of the adolescents.

Our findings must be viewed within the context of the study's limitations. An important limitation, inherent in every disaster study, is that participants knew the purpose of the study since it was explained in the consent form. As a result, it is conceivable that the evacuees gave responses that they believed were expected of them, thus magnifying the disparities in health perceptions among the groups. However, the differences in self-rated health reflected less positive ratings rather than perceptions at the negative end of the scale. Indeed, the percent rating themselves as poor/very poor in all three groups (5.0-8.6%) was lower than the 13.3% rate found in a national sample of 18-29 year olds in Ukraine [10]. Another limitation is that the study was conducted in one location, and we do not know how well the findings would generalize to evacuees resettled elsewhere. Unfortunately, as often happens in the chaotic aftermath of a disaster, there is no comprehensive listing of where all of the evacuees were resettled. We note here though that the results were consistent with findings for evacuees who emigrated to Israel [5]. Perhaps the most crucial limitation is that the early and most horrifying events unleashed by the accident were not directly experienced by the adolescents. However, they have been exposed to rumors and public concerns about their health throughout their lives [2,31]. A related limitation is that all three groups of adolescents and their mothers were exposed to stressors from the Chornobyl accident by virtue of living in Kyiv. Indeed, in the summer of 1986, Kyiv was a city without children as youngsters were sent away to live with relatives because of fears about radioactive contamination. Although Kyiv in fact received far less radiation than other areas of Ukraine and Belarus, we found that in 1997, three-quarters of both evacuee and classmate mothers believed that Kyiv was not a safe place to live [3]. Thus, we do not have a true non-exposed comparison group. On the other hand, one could argue that a truly unexposed sample from another region would not constitute a valid control group because they would not be drawn from the same source population with the same set of values and life experiences. There may also have been a bias for those exposed children and/or their mothers who were more anxious about the health effects of Chornobyl to participate in our study. Families who have already been reassured by frequent participation in multiple earlier screenings may have been uninterested in taking part in yet another study. Lastly, we cannot disentangle cause and effect in this study, and thus we are careful about the inferences we draw from our results.

Taken at face value, the impact of the Chornobyl disaster on self-rated health and medical illness suggests that the widespread fears among the population about the health consequences of the disaster have been absorbed by the very cohort at the heart of all the attention. Partly these fears have been confirmed by the increase in thyroid cancer among evacuees exposed at a young age [32]. In our sample, four evacuees had their thyroid removed in contrast to only one control. In addition, almost one-quarter of the evacuees reported being diagnosed with a thyroid problem in the 5 years before interview; and almost one in five were found to have enlarged thyroids upon examination. Thyroid enlargement has been a relatively common finding in studies of Chornobyl exposed children. For example, Hatch et al. [33] found a rate of 17% in an exposed cohort at a similar age. However, it does not appear to be related to radiation exposure [33] and may instead be a reflection of the moderate iodine deficiency known to occur to a greater degree in the Chornobyl area than in the city of Kyiv [34]. It is possible that being given a diagnosis of benign thyroid enlargement in this or earlier health-monitoring programs serves as a constant reminder to the evacuees of their ongoing increased risk for thyroid cancer, contributing to their perception of poor health. We found only a small number of thyroid nodules in our sample using thyroid palpation; thyroid sonography is a much more sensitive method to detect thyroid nodules and its use in other studies of Chornobyl evacuees undoubtedly accounts for the higher prevalence of nodules found in those evaluations [33].

Another reason the evacuees have absorbed the concerns about their health is that they have had multiple hospitalizations. Indeed, one-third of evacuees were hospitalized two or more times in the past five years compared to one-fifth of the controls. Many of these hospitalizations were for physical check-ups which yielded a surplus of diagnoses, including vascular dystony. Stiehm [21] examined a sample of children hospitalized in Kyiv with this diagnosis and concluded that by western classification, they had no diagnosable conditions. Moreover, Mikolajczyk et al. [7] found that having 2+ visits to a doctor was significantly associated with increased psychosomatic complaints in a study of university students in Germany, Poland, and Bulgaria. Thus, the evacuee adolescents have understandable concerns about their health, and it is a testament to their resilience that only a handful actually described their health as poor.

The pattern of results for the epidemiologic risk factors, particularly female gender, and poorer material circumstances, self-esteem, and family communication, confirmed findings from youth and university students conducted in other, mostly Western, countries. In addition, consistent with the adult literature, adolescents who believed that their health was adversely affected by the accident reported less satisfactory health and more illnesses. The causal sequence in this case cannot be determined. One finding that was discrepant from previous studies was the more negative perceptions of health by the mothers in comparison with their children and the surprisingly strong associations between mother-child ratings given that their children were 19 years old. In some cultures, parental influence is markedly diminished in late adolescence, and one would expect to find smaller and perhaps non-significant associations between mother-child pairs at this age. Not only was this not the case in our sample, but we replicated the association in all three groups. The Chernobyl Forum recommended that renewed efforts at risk communication be initiated to provide the community with accurate information about the health consequences of the accident [35]. Our data suggest that such programs should be directed at the family unit rather than at parents or adolescents separately.

When the children were 11 years old, we hypothesized that hospitalizing them for periodic check-ups could create a sense of learned helplessness [17]. However, we are now struck by the resilience of the evacuees in the face of public concerns about thyroid cancer, their hospitalization experiences, the diagnoses they received, and their mothers' concerns about their health. One explanation is that they did not experience the catastrophe in real time and like teenage disaster survivors elsewhere, they therefore perceived themselves as 'invulnerable' [36]. Indeed, teenagers generally see themselves as invincible [37,38]. It is also possible that they have adapted to seeing doctors frequently, and the impact on their self-assessments, while significant, has blunted its negative valence. Thus, as a group, they describe their health in less satisfactory but not outright negative terms. At the focus groups we conducted prior to the fieldwork, we first met with 10 mothers (7 evacuees; 3 from Kyiv) who derailed the discussion within the first five minutes and talked non-stop about their children's health problems due to Chornobyl. In contrast, their 10 adolescent children had little interest in either Chornobyl or their health, and even commented that "it's our mothers' issue, not ours." The question that remains to be addressed is whether the differences in perceived health have spilled over in producing differences in mental health, particularly depression and anxiety.

## Conclusion

The findings showed that evacuee adolescents rated their health less favourably and reported more medically diagnosed illnesses than their peers in Kyiv. The findings were consistent with results from studies of adults sampled after the accident from high exposure regions. Epidemiologic, disaster-related, and maternal risk factors independently influenced the adolescents' subjective health evaluations, particularly being hospitalized multiple times, being female, health risk perceptions, and negative maternal health perceptions.

## Competing interests

The authors declare that they have no competing interests. Drs. Harold Carlson and Gabrielle Carlson have received funds for their work on the treatment of bipolar disorder from Bristol Myers Squibb, Otsuka, Glaxo Smith Kline, Lundbeck, Validus, McNeil, Shire and Eli Lilly Corporation, but these organizations do not stand to gain or lose financially by publication of this paper.

## Authors' contributions

EJB was the principal investigator of the project and drafted the manuscript. DPT supervised the medical data coordination between Stony Brook and Kyiv and assisted with drafting the manuscript. LTG performed the statistical analyses and made substantial contributions to drafting the manuscript. JAB trained the physicians, oversaw the medical and laboratory work, and drafted sections of the manuscript. JMH participated in the design and conceptualization of the study, the monitoring the field work, and made critical revisions to the paper. SFG created the structure for the medical examinations and made revisions to the manuscript. HC made critical contributions to the interpretation of the blood test and examination findings and to the writing of the manuscript. GAC participated in the design and conceptualization of the study, choice of measures, and made critical revisions to the manuscript.

All authors read and approved the final manuscript.

## Pre-publication history

The pre-publication history for this paper can be accessed here:

http://www.biomedcentral.com/1471-2458/9/417/prepub
